# Discovery and assembly of repeat family pseudomolecules from sparse genomic sequence data using the Assisted Automated Assembler of Repeat Families (AAARF) algorithm

**DOI:** 10.1186/1471-2105-9-235

**Published:** 2008-05-13

**Authors:** Jeremy D DeBarry, Renyi Liu, Jeffrey L Bennetzen

**Affiliations:** 1 Department of Genetics, University of Georgia, Athens, GA 30602-7223, USA; 2 Current Address: Department of Botany and Plant Sciences, University of California, Riverside, CA 92521, USA

## Abstract

**Background:**

Higher eukaryotic genomes are typically large, complex and filled with both genes and multiple classes of repetitive DNA. The repetitive DNAs, primarily transposable elements, are a rapidly evolving genome component that can provide the raw material for novel selected functions and also indicate the mechanisms and history of genome evolution in any ancestral lineage. Despite their abundance, universality and significance, studies of genomic repeat content have been largely limited to analyses of the repeats in fully sequenced genomes.

**Results:**

In order to facilitate a broader range of repeat analyses, the Assisted Automated Assembler of Repeat Families algorithm has been developed. This program, written in PERL and with numerous adjustable parameters, identifies sequence overlaps in small shotgun sequence datasets and walks them out to create long pseudomolecules representing the most abundant repeats in any genome. Testing of this program in maize indicated that it found and assembled all of the major repeats in one or more pseudomolecules, including coverage of the major Long Terminal Repeat retrotransposon families. Both Sanger sequence and 454 datasets were appropriate.

**Conclusion:**

These results now indicate that hundreds of higher eukaryotic genomes can be efficiently characterized for the nature, abundance and evolution of their major repetitive DNA components.

## 1 Background

All higher eukaryotic genomes are rich in multiple classes of repetitive DNA. Transposable elements (TEs) are particularly abundant, and are the most important factor responsible for genome size variation in both animals and plants [1]. Although TEs have been judged to be 'junk DNA', existing within host genomes as purely selfish denizens [2,3], it has been found that some repetitive elements perform important roles in their host genomes [4-7], for instance, as with the telomere-generating Het-A and TART retroelements of Drosophila [8]. In the last decade, the generation of whole genome sequence data from multiple species has provided the opportunity to investigate the relative contributions of repetitive elements to genomic organization and evolution [9-14].

TEs have been identified in nearly all organisms studied to date. They are reported to account for 3% of the 4 Mb yeast genome [6], 20% of the 140 Mb Arabidopsis genome [13], 22% of the 165 Mb Drosophila genome [15], 35% of the 390 Mb rice genome [14], 15% of the 1200 Mb chicken genome [16], > 60% of the 2400 Mb maize genome [17], 46% of the 3200 Mb human genome [18], > 70% of the 4800 Mb barley genome [19] and > 90% of the 16 Gb wheat genome [20].

One type of TE is the Long Terminal Repeat (LTR) retrotransposon (LRP). LRPs account for the great majority of the repetitive DNA in plant genomes [19]. LRPs are named for the LTRs that flank the coding regions of the element. LTRs contain regulatory sequences important for the proper expression of the LRP, such as the transcription start site and polyadenylation signals. Located between the LTRs of the element are the coding regions that provide the protein products necessary for the element's transposition. LRPs typically contain two open reading frames (ORFs). The first of these, gag, contains products necessary for the formation of a virus-like particle where reverse transcription of the RNA intermediate takes place. The second ORF, pol, contains the protease, reverse transcriptase, RNase-H and integrase regions necessary for element protein processing, reverse transcription, degradation of the RNA intermediate and integration into a new genome location [6,19].

Historically, LRPs, other TEs and other types of repetitive DNA have been identified in a genome by their presence in or near genes, or by the amplification of sequences with homology to TEs from other species [11,21]. Due in part to the wealth of sequence information provided by whole genome sequencing projects, the opportunities to detect TEs have greatly expanded in recent years. However, the high cost of completed whole genome sequencing makes this approach inappropriate to investigate the TE content of a large number of species.

As interest in TEs and other repetitive elements has grown, techniques have been developed to discover and investigate them directly. Without the availability of a large amount of assembled genome sequence, studies focused on the identification of TEs within a genome have been restricted to the use of hybridization and PCR techniques [6,22-25]. While these methods are useful for the identification of repeats that are highly homologous to already-discovered repeats, they lack the power necessary to discover or precisely quantitate new classes of repetitive DNA. Sample sequence analysis, wherein a small amount of DNA sequence is generated from randomly selected clones [26-28], can efficiently provide unbiased genomic information, that could potentially be analyzed for repetitive DNA content. The programs RECON [29] and ReAS [30] have been designed for the *de novo *discovery of repeats. RECON utilizes assembled genomic sequence as input, while ReAS was designed for highly redundant genomic coverage with Sanger sequence data sets [30]. ReAS was found to not be adaptable for use with 454 sequence data [31]. Thus, there is currently no method available that is designed to discover and describe genomic repeats using small quantities of unassembled Sanger or 454 sequence data.

In order to provide an automated method for the efficient characterization of all of the high copy number repeats within a genome from sparse sample sequence data, the Assisted Automated Assembler of Repeat Families, or AAARF, algorithm is described in this article. Tests of AAARF on the *Zea mays *genome, using random shotgun sequence data from a Sanger sequencing output and from a simulated 454 sequence data set are presented. The *Z. mays *genome has been well studied in terms of repeat content and provides an excellent opportunity to test AAARF's effectiveness. For both data sets, the program constructed builds representing repeats necessary for genome structure and function (centromeric, ribosomal and knob repeats) and the seven most abundant LRPs in the *Z. mays *genome.

## 2 Implementation

### 2.1 The AAARF algorithm

**Figure 1:**
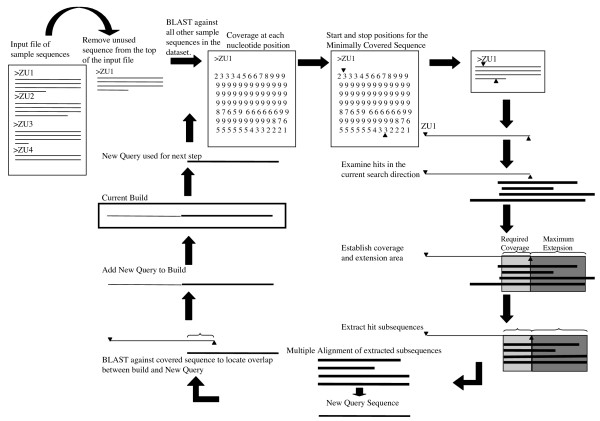
**Schematic of the AAARF algorithm.** Shown is an illustration of the AAARF process used to assemble builds representing high copy number repeat families from sample sequence data.

AAARF works by comparing sample sequences from a genome to one another via BLAST [32] and then using a series of BLAST analyses and multiple alignments to "walk out" an *in silico *produced molecule, or "build", that represents a discreet family of repeats from the target organism. A schematic of the AAARF process is shown in Figure 1. Initially, AAARF accepts a fasta file of sample sequences as input. An unused sequence (the first in the input file in Figure 1) in the dataset is BLASTed against all other sample sequences. Next, a coverage matrix representing the detected similarities for the sequence is generated based on this BLAST output. The coverage matrix is a representation of the coverage depth for each nucleotide position in the sequence being considered. The coverage matrix is used to assess the repetitive nature of the sample sequence. The program calculates start and stop points for the sequence that represent the boundaries of a user defined minimum coverage threshold, based on a minimum depth of coverage requirement. This section of the sequence is known as the Minimally Covered Sequence, or MCS. If this sequence doesn't meet the minimum coverage requirement, or the MCS is too short, the sequence is rejected and the process starts again with the next sequence in the dataset. The portion of the sample sequence that corresponds to the MCS is extracted from the sample sequence. Next, the BLAST output is searched to locate sequences that overlap the MCS in the current search direction (right, in Figure 1). Sequences that meet the minimum coverage requirements and have the potential to extend the process are selected. A sub-sequence corresponding to the coverage and extension criteria are extracted from the chosen sequences. A multiple alignment of the selected sequences is performed to generate a consensus sequence called the New Query (NQ). The NQ is BLASTed against the MCS to locate the overlap region. Such an overlap indicates that the multiple alignment has produced a sequence that is able to extend the build process correctly. The overlap region is trimmed from the MCS and the NQ is added to the growing build. To complete a single step, the NQ re-enters the loop and is BLASTed against the sample sequence dataset. Directional extension will continue until either there is insufficient evidence in the coverage matrix to indicate a repetitive sequence or the requirement for a minimum number of sequences to extend the process is not met. Extension then begins from the same starting sequence in the opposite direction. Sample sequences are only allowed to participate in a single build. This prevents the seeding of additional builds by sequences already used to construct a build. A sample sequence can participate in both directional extensions of a build in an effort to produce a build that most closely represents a full-length element family. The size of a single round of extension can be smaller than an individual sample sequence. To ensure that an entire sample sequence is used in build construction, the user can adjust the number of times that a sequence can be used to extend the build in a given direction. Using the program output along with the sample sequence dataset under investigation, it is possible to locate the biological ends of the elements represented by the builds.

### 2.2 Program construction input and output

AAARF consists of a single script written in the PERL programming language (see Additional files 1 and 2). The program makes use of a suite of freely available BioPerl modules [33]. BLAST and Clustalw [34] are used for sequence comparisons and multiple alignments, respectively. A single file of fasta sequences and a BLAST database made from the same sequences, are used as input to the program. The program produces a fasta file of builds and a diagnostic log file. The log file is produced with the Log4Perl module [35], a customizable PERL logger. AAARF's activities during the build process are recorded in this file for later inspection. AAARF was run on a 2 GHz Macintosh with 1 Gb of RAM. Analysis of the Sanger sequence dataset test was completed in 302 minutes (10,000 sequences), and analysis of the simulated 454 dataset was completed in 1.5 days (50,419 sequences).

### 2.3 Build issues

There are a number of potential issues that arise when attempting to construct full-length repetitive elements from sample sequence data. It is important that these concerns are understood in order to properly implement the AAARF algorithm and interpret its results. The first of these deals with the internalization and single copy nature of LTRs within a build. LTRs are arranged at either end of a LRP in a direct repeat orientation. Because AAARF utilizes sequence similarity to construct builds, the two LTRs of a LRP will usually be combined into a single LTR composed of sample sequences from either end of multiple elements. Since it is unlikely that both LTRs of a single LRP will be present in a random set of sample sequences, a build's LTR region will likely be a combination of LTRs from multiple related LRPs. Also, because the construction of a build begins at a random point along a repeat (as dictated by the randomly chosen sample sequence that initiates the build), the LTR region will most likely not be present at the end of the build, but internalized within the build.

The next issue to consider is the size of the builds as compared to actual repeats. AAARF builds may be larger than the corresponding type of native repeat. It is possible that a full-length copy of the element may be produced for both search directions for a single build. The number of times that a sequence is allowed to participate in a build are restricted in a given search direction based on the sizes of the sample sequences use as input (Sanger or 454). The count is reset at the start of each directional search, allowing for multiple full-length representations to be present in a single build. This restriction on the number of times that a sequence is used in a search direction ensures that an entire sample sequence is only used once in each build direction. The parameter controlling the number of times that a sequence can be used to extend a build in a single direction is adjustable by the user.

Another way that builds may exceed the size of actual repeats reflects possible size differences within the repeat family. Some families will contain members that are larger than others due to insertions or deletions in certain family members. As long as the affected member is able to replicate, it will be maintained in the genome, and if it is present at a sufficient copy number, be incorporated into a build. Thus, it is possible that a build may represent the largest members of a family, while the smaller members are also contained within the build.

Alternatively, builds may be smaller than native repeats. In general, it is likely that a build will be shorter than a native LRP by the length of one LTR because of LTR internalization. A small build will also be produced in any situation where there are not enough sequences present for a particular type of repeat to be assembled by the program. For instance, LTRs by their nature should be present in numbers at least 2× the amount of sequence for any other region of the element. Since they are present more frequently in the dataset, it is possible that builds representing the LTRs of a family of elements will be produced in cases where construction of the internal regions of the elements is not possible. Also, a build may be broken up due to indels in some family members. Indels can be incorporated into a build as long as there are enough sequences in the dataset to cross the indel-generated build gap. However, if the indel is large enough that it causes the depth of coverage to drop below the requisite threshold, build construction will stop in that direction.

Repeat families may contain levels of sequence diversity that make it impossible for AAARF to assemble all members into a single build. In this case, it is possible that the family will be broken up into multiple builds. For nearly all repeat families that AAARF was able to construct in full-length or near full-length form, there were multiple builds for each family (Table 1). Because of the issues discussed above, it is possible for a build to be fragmented due to low coverage. If this is the case, then there will be no full-length build for a particular family. Rather, the builds for that family will be present in fragmented form. It is also possible that sample sequences from regions that differ between members of the same repeat family may initiate their own build. In this instance there may be a full-length build for a family and additional builds representing regions that were unable to collapse into the full-length build. In some cases it is possible to resolve build fragmentation issues and identify builds that belong to the same family. Shared sequence similarity among AAARF-produced builds can be used to infer relationships between builds that were not combined because of sequence divergence issues or positional effects of sequence used in the build process. For the three fragmented builds in the Sanger and 454 tests, comparison of the builds to one another was able to successfully resolve one of the fragmentation events.

**Table 1:** Sanger and 454 results compared to the seven most abundant LTR retrotransposon families in maize

**Most Abundant LRPs in Maize (a)**	**Percent Genome Composition**	**Element Size (kb)**	**LTR Size (kb)**	**Sanger: Best Build Size (bp)**	**Sanger: Number of Builds**	**454: Best Build Size (bp)**	**454: Number of Builds**
*Huck*	10.7	11–14	1.6	23706 (FL)	8	11269 (F)	10
*Ji*	9.4	8.5–10	1.3	10041 (FL)	4	9432 (FL)	3
*Opie*	7.1	6.5–9	1.3	8150 (FL)	3	9599 (FL)	2
*Zeon*	4.8	7.3	0.6	7412 (FL)	7	1225 (I)	4
*Grande*	3.9	10.5–13.5	0.6	8469 (F)	4	6459 (I)	3
*Cinful*	3.5	8.5	0.6	7264 (F)	5	1600 (I)	4
*Xilon ***(b)**	3.1	11.7	2.7	8971 (I)	1	2107 (I)	5

**(a) **Percent genome composition data from Meyers et al. 2001.

**(b) ***Xilon *percent genome composition from Meyers, pers. comm.

FL = Builds representing full-length copies of repeat families, F = Builds representing fragmented copies of repeat families, I = Incomplete builds

## 3 Results

### 3.1 Testing the AAARF algorithm

A wealth of information exists regarding the repeats found in the maize genome (Table 1), particularly the LRP content [17,26,36-39]. The maize genome is approximately 2400 Mb in size [17], and > 60% of the genome is composed of repetitive DNA [40], primarily LRPs [6]. In order to ascertain AAARF's effectiveness, a database of known maize genomic repeats was assembled. This database is a combination of TIGR's maize repeat database [41] and a maize repeat database developed by P. San Miguel at Purdue University (P. San Miguel, pers. comm.). Each of these databases contains the sequences of many different repeat families and individual family members found in maize. Builds produced by AAARF were BLASTed against this known repeat database to investigate how accurately the builds represent actual genomic repeats and to examine how well the program constructs builds representing distinct families of repeats.

To classify a build as representing a type of known repeat, several criteria were used. A build was required to be at least 1 kb in size, and to have at least one hit with a minimum BLAST score of 100 when compared to the known repeat database. A score of less than 100 was taken as evidence that no useful sequence similarity existed between the build and the known repeat database. Builds were also inspected to ensure that they did not improperly fuse two repeat families. Finally, each build was examined to ensure that it showed similarity to a single family of known repeats over at least 90% of its length. In this regard, all builds generated by AAARF from the maize data analyzed (below) were found to be homologous to an already-known maize repeat family, indicating both the quality and comprehensiveness of the TIGR and San Miguel databases.

The ultimate goal of the AAARF algorithm is to construct the best build possible for a given family of repeats. How well AAARF is able to accomplish this is dependent on the amount of sequence in the sample data set for a given repeat family. There are many issues regarding the build process that were considered in the examination of the builds (discussed in Implementation). A build was classified as full-length only if it showed similarity to the entire length of multiple members of a single discreet repeat family.

### 3.2 AAARF analysis of Sanger sample sequence data from maize

Random unfiltered shotgun sequence reads produced by Sanger sequencing for maize are available from TIGR [42]. Sequences were obtained from TIGR in December of 2005. We selected the first 10,000 available sequences from this database for input into AAARF. This sample sequence dataset totaled 7,821,671 bp (average read size 782 bp), representing 0.33% of the maize genome (Table 2). Input sequences were screened for vector content using NCBI's UNIVEC database[43]. AAARF produced 180 builds from the Sanger sample sequence dataset described above (Table 2) and the parameter set described in Table 3. Of these, 57 were chosen for further analysis (Table 2). As expected, AAARF assembled builds for non-TE repeats, including centromeric repeats, ribosomal repeats and knob repeats. In addition, builds representing all 7 of the most abundant LRP families in the maize genome were constructed (Table 1). Full-length builds were constructed for the four most abundant families. Builds representing the *Grande *and *Cinful *families were fragmented, such that there was a region missing from each build. For both *Grande *and *Cinful*, the missing region was assembled intact in additional builds for each family. For the *Xilon *build, a 700 bp portion of the LTR region found in native *Xilon *elements was missing. Thus, AAARF constructed full-length or near full-length builds representing the seven most abundant LRP families in the maize genome. In all cases, AAARF was able to assemble a build for each of these families that readily identified the repeat family. For the *Grande *family (the build with the largest missing fragment) the largest build covered 80% of the expected family size.

**Table 2:** Overall results of AAARF tests of Sanger and simulated 454 data sets

	**Number of Sample Sequences**	**Sequence Amount (bp) (%Genome)**	**Total Builds**	**Builds < 1 kb (a)**	**Fused Builds (a)**	**Build Coverage < 90% (a)**	**No Hits/Score Too Low (a)**	**Analyzed Builds**
**Sanger Sequence Build Results**	10000	7821671 (0.33%)	180	46	5	49	23	57
**454 Sequence Build Results**	50419	5045000 (0.21%)	63	2	2	12	1	46

**(a) **Builds not further analyzed

**Table 3:** Parameter settings for Sanger and 454 tests

	**Minimum Hit Length**	**Minimum Hit Identity**	**Maximum e-value**	**Required Length of MCS**	**Required MCS Coverage Depth**	**Minimum Number of Hits for Extension**
**Sanger**	150	89	1.00E-25	150	3	2
**454**	30	88	1.00E-10	30	3	2

	**Required Coverage Length**	**Maximum Extend Length**	**Minimum BL2SEQ Hit Size**	**Maximum BL2SEQ e-value**	**Maximum Number of Times a Sequence Can Be Used in One Direction**	**Other**

**Sanger**	150	50	90	1.00E-10	13	
**454**	30	40	15	10	4	BL2SEQ Word Size: 7

### 3.3 454 sequence analysis

454 sequence analysis [44] is an emerging high throughput technology that greatly lowers the cost of data generation. This will facilitate the generation of sample sequence datasets for a wide array of species. The initial 454 sequences had an average length of ~100 bp [44]. In order to test the ability of AAARF to utilize this type of data, a simulated dataset of 454 sequences for the maize genome was generated (see below). Sequences were screened for vector content using NCBI's UNIVEC database [43]. A total of 50,419 sequences representing 5,045,000 bp (average read size 100 bp) were used as input. This dataset represents ~0.21% of the maize genome (Table 2).

The same database of known repeats and the same build classification criteria used for testing the output of the Sanger sequence test were used for analyzing the 454 sequences. Smaller input sequences cause a variety of build issues stemming from the required BLAST parameter settings used by AAARF. Because of the reduced size of the input sequences compared to the previous dataset, the number of builds with a total size of less than 1 kb was greatly increased. Since AAARF only allows each sample sequence to participate in a single build, builds of less than 1 kb in length were rejected. This ensured that as many sequences as possible were available to the program for each build, instead of being utilized in smaller, ultimately uninformative builds.

Despite the inherent difficulties posed by shorter sequences, and the overall reduction in sample sequence dataset size, AAARF generated 46 builds that were identified as belonging to known repeat families (Table 2). All *Huck *family builds were fragmented in the output, with an approximately 1.4 kb fragment missing from the largest build. This fragment was present intact in an additional *Huck *build. *Ji *and *Opie *were constructed in full-length form. For the remaining 4 families in Table 1, only *Grande *was constructed in a large build. It is possible that the inability of the program to construct the other four elements in a full-length size is due to the 454 sample sequence data being only ~64% the size of the Sanger sequence dataset. It is also possible that further parameter optimization for 454 data will yield superior results. As 454 sequencing technology continues to develop, the average size of the reads is increasing. Such an increase in sequence size will facilitate their use as input sequences for the AAARF approach.

Apart from sequence size issues, 454 technology brings with it a new set of issues with regard to reliability statistics and error rates [45]. In particular homopolymers pose specific problems due to the nature of the pyrosequencing technology that 454 sequencing employs [46]. Precise parameter adjustments to account for these issues can be made with the use of actual 454 data.

### 3.4 454 dataset construction

In order to simulate a 454 sequence dataset for maize, all available unfiltered shotgun sequences for the maize genome were downloaded from TIGR [42]. At the time of this analysis, there were 50,877 shotgun sequences in this dataset. The data were divided into three subsets of 16,959 sequences each. To simulate an average read size of 100 bp, a subsequence of each read was extracted. Positions 100–150, 100–200 and 100–250 were extracted from all sequences in each set respectively. The extraction was initiated from the 100 bp position for each read to avoid any possible sequencing errors at the end of the read. Only one sequence was extracted from each shotgun read to provide a random sampling. This produced three datasets composed of 50, 100 and 150 bp sequences. For each of the three subsets, there were sequences that were not large enough for the extraction process, resulting in a final count of 50,419 sequences.

### 3.5 Parameters

AAARF parameters for the Sanger sequencing and 454 datasets were determined by trial and error in order to examine how changes in the program parameters affected program output. Adjustable parameters for both tests are found in Table 3. Parameters affecting the required length of BLAST hits, coverage, extension length and maximum number of times that a sequence is allowed to participate in a search direction were chosen based on the sizes of the sample sequences used for each test. Identity and e-value requirements for both tests were determined by trial and error.

Required depth of coverage for a sequence to be classified as repetitive, and the required minimum number of sequences for extension were chosen based on an interpretation of what was necessary to recognize a repetitive sequence. The presence of a particular sequence in the sample sequence dataset at least 3 times was seen as evidence of its repetitive nature. Because of slight positional variation of the coordinates of sequences participating in the AAARF process, it is possible that a sequence that belongs in a build may be rejected due to a difference with required positional parameters generated during MCS construction. In order to account for this phenomenon, only 2 sequences were required for extension. This did not affect the accuracy of the builds when compared to a test requiring a 3 sequence minimum for extension.

For the 454 test, the word-size BLAST parameter for the BL2SEQ was lowered to 7 from the standard 11. During testing it became apparent that the small size of the 454 sequences presented problems with the detection of overlap between the New Query Sequence and the MCS (Figure 1). Reduction in the required word size facilitated overlap detection.

### 3.6 End finding

As the name of the program indicates, there is a hands-on component to the AAARF process. The program assembles builds representing discreet families of genomic repeats while maintaining the correct order and orientation of the elements. However, the element components are unlikely to be placed in the same end-to-end fashion as a typical element. This is due to the random starting point for a build. To alleviate this issue, a method for the identification of the biological element endpoints of LRPs for AAARF-produced builds has been developed.

**Figure 2:**
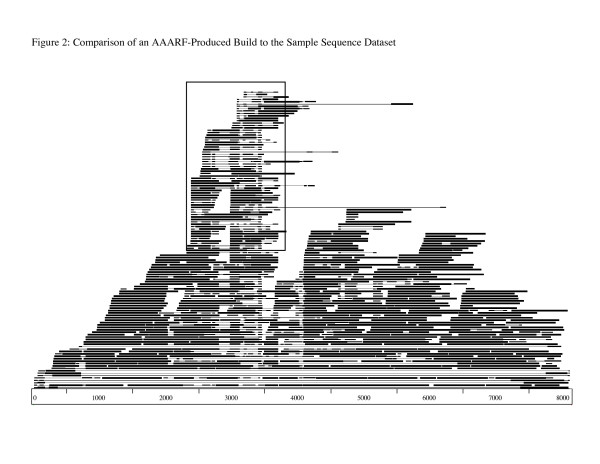
**Comparison of an AAARF-produced build to the sample sequence dataset.** Shown is the BLAST result of an AAARF-generated build compared to the sample sequence dataset used to create it. The bottom, metered line represents the full-length *Opie *build (8,150 bp) from the Sanger sequence test. Smaller lines above represent sample sequences. Regions of shared similarity between the build and the sample sequences are indicated by the position of the sample sequences relative to the build. A region of increased coverage on the build, combined with sample sequences whose similarity to the build stops at the same position (boxed area), indicates the likely presence of an LTR.

For LRPs, the ultimate goal is to locate the LTR region of the build as this region contains both element ends. Initially, BLASTx is used to locate possible protein coding regions within the build, to narrow the area of the build where the LTR may be found. Next, the build is used in a BLAST analysis against the sample sequence data set that was used as input for the AAARF program. This BLAST provides coverage information for the build. In addition to facilitating the location of the biological endpoints of the build, this information can be compared to the AAARF-generated diagnostic log file to ensure that all suitable sequences were used to construct a given build. If sequences are found in this comparison that were not used to construct a build, program parameters can be altered to incorporate these sequences. The Apollo program [47] was used to visualize this comparison (Figure 2). Using this information, it is possible to examine the build for regions that are represented at a greater depth of coverage in the sample sequence dataset (Figure 2). The region of the build that contains the LTR should be covered by sample sequences at least twice as deeply as the rest of the build. This is due to the presence of LTRs at either end of a native full-length element and the solo-LTRs present in the genome as a result of partial element removal by unequal recombination. Around this region of increased coverage, the individual sample sequences that cover the region are inspected to locate sequences that are truncated at approximately the same position on the build (Figure 2). Because the LTRs are present on either side of a native full-length element, sample sequences that include the LTR boundaries may include either sequence from the interior of the element or sequence from the genome surrounding the element.

Once these possible boundaries are identified, this region is extracted from the build for further inspection. There are four possible orientations for the LTR region, depending on the orientation and strandedness of the sequence that initiated the build. The extracted section is then inspected for conserved LTR terminal dinucleotide motifs and the Primer Binding Site and Polypurine Tract for the element family that has been constructed. These structures are localized to the LTR region of native LRPs and can be used to indicate the presence of an LTR within the build. For a build representing a full-length element, this information can then be used to manually reconstruct a build with LTRs at either end of the build. For builds that are less than full-length, this approach will be useful in identifying the LTR region if it is contained in the build. As a proof of principle, this method was used to identify biological element endpoints for the full-length *Opie *build in the Sanger sequence test. The location of the LTR within the build was verified using actual elements from the known repeat database.

## 4 Discussion

AAARF provides an excellent resource for the initial characterization of high copy number repeats in a genome that has been subjected to very limited shotgun sequence analysis. Because of the nature of the AAARF process, the pseudomolecules it produces are "patchwork" representations of native repeat elements. A multiple alignment of selected overlapping and extending sequences is used for each directional extension. Thus, each step represents sections from actual repeats found in the target genome. Sequence divergence information for actual elements is available via the sample sequences used to construct the pseudomolecule. By comparing these pseudomolecules to the input sample sequence dataset, information about the evolutionary history of the assembled repeat family and the percent of the target genome composed of that repeat can be determined.

While these tests have focused on AAARF's ability to construct builds representing LRP sequences, the utility of the program extends to the construction of builds representing any high copy number repeat in a genome. As long as there is sufficient sequence in the sample sequence dataset to represent the repeat, AAARF will construct a build for it.

The parameters used here were developed for use in the maize genome. Depending on the type of sample sequence being used or the species being investigated, it will be necessary to alter the program parameters to produce the most accurate builds possible. This is the primary reason that the diagnostic test log is produced as a part of the AAARF process. Using this log file, it will be possible to optimize the parameter set for any species under investigation.

## 5 Conclusion

As understanding of the prevalence and effects of LRPs has increased, it has become apparent that understanding the evolutionary dynamics of LRPs both within individual genomes and among different species is necessary for a complete understanding of genome structure and history. The true utility of the AAARF approach is its ability to facilitate such an understanding. Because AAARF is designed to function on sample sequence data, important information about the TE content of a genome can be investigated with a small amount of sequence, making this type of analysis feasible for studies that involve hundreds of species.

## 6 Availability and requirements

• **Project Name: **Assisted Automated Assembler of Repeat Families

• **Project Home Page: **

• **Operating System: **Mac OS X

• **Programming Language: **Perl

• **Other Requirements: **Bioperl 1.2.3 or higher, Bioperl Run Package 1.4 or higher, Log4perl 1.01 or higher, NCBI BLAST 2.2.9 or higher, Clustalw 1.8.3 or higher

• **License: **GNU Lesser General Public License

• **Restrictions: **none

## 7 Authors' contributions

JDD co-designed the algorithm, conducted the testing and implementation of the algorithm and drafted the manuscript. RL co-designed the algorithm and reviewed the manuscript. JLB conceived the approach, participated in its design, provided valuable guidance and critically edited the manuscript. All authors read and approved the final manuscript.

## Supplementary Material

Additional file 1 AAARF Algorithm Perl Script. This is the AAARF algorithm. The file can be viewed in a text editor. Examples are BBedit for Macintosh and Wordpad for Windows. This file is also available at the project homepage . Click here for file

Additional file 2 AAARF Program Documentation. This is the AAARF algorithm documentation. The file is intended to aid in the use of the program and provides instructions for its use. The file can be viewed in a text editor. Examples are BBedit for Macintosh and Wordpad for Windows. This file is also available at the project homepage . Click here for file
